# Long Non-coding RNA Regulation of Mesenchymal Stem Cell Homeostasis and Differentiation: Advances, Challenges, and Perspectives

**DOI:** 10.3389/fcell.2021.711005

**Published:** 2021-07-22

**Authors:** Yanlei Yang, Suying Liu, Chengmei He, Zhilei Chen, Taibiao Lyu, Liuting Zeng, Li Wang, Fengchun Zhang, Hua Chen, Robert Chunhua Zhao

**Affiliations:** ^1^Key Laboratory of the Ministry of Education, Department of Rheumatology and Clinical Immunology, Clinical Immunology Center, Peking Union Medical College Hospital, Chinese Academy of Medical Sciences, Peking Union Medical College, Beijing, China; ^2^Beijing Key Laboratory (No. BZO381), School of Basic Medicine, Center of Excellence in Tissue Engineering, Peking Union Medical College Hospital, Institute of Basic Medical Sciences, Chinese Academy of Medical Sciences, Peking Union Medical College, Beijing, China; ^3^School of Life Sciences, Shanghai University, Shanghai, China

**Keywords:** mesenchymal stem cells, long non-coding RNAs, differentiation, homeostasis, exosomes

## Abstract

Given the self-renewal, multi-differentiation, immunoregulatory, and tissue maintenance properties, mesenchymal stem cells (MSCs) are promising candidates for stem cell-based therapies. Breakthroughs have been made in uncovering MSCs as key contributors to homeostasis and the regenerative repair of tissues and organs derived from three germ layers. MSC differentiation into specialized cell types is sophisticatedly regulated, and accumulating evidence suggests long non-coding RNAs (lncRNAs) as the master regulators of various biological processes including the maintenance of homeostasis and multi-differentiation functions through epigenetic, transcriptional, and post-translational mechanisms. LncRNAs are ubiquitous and generally referred to as non-coding transcripts longer than 200 bp. Most lncRNAs are evolutionary conserved and species-specific; however, the weak conservation of their sequences across species does not affect their diverse biological functions. Although numerous lncRNAs have been annotated and studied, they are nevertheless only the tip of the iceberg; the rest remain to be discovered. In this review, we characterize MSC functions in homeostasis and highlight recent advances on the functions and mechanisms of lncRNAs in regulating MSC homeostasis and differentiation. We also discuss the current challenges and perspectives for understanding the roles of lncRNAs in MSC functions in homeostasis, which could help develop promising targets for MSC-based therapies.

## Introduction

Mesenchymal stem cells (MSCs) are heterogeneous, multipotent adult stem cells that originate in the mesoderm and that have been isolated from diverse tissues such as adipose tissue, bone marrow, and umbilical cord. Due to their self-renewal, multilineage differentiation potential, extensive immunomodulatory effects, and tissue maintenance properties, MSCs have emerged as attractive tools for cell-based therapies and have been involved as treatment options for hematological diseases, autoimmune diseases, and peripheral nerve injuries (Chen et al., 2019; Yousefi et al., 2019; Zoehler et al., 2020). Currently, there have been breakthroughs in uncovering MSCs as key contributors to homeostasis and the regenerative repair of tissues and organs derived from three germ layers (mesoderm, ectoderm, and endoderm) (Sui et al., 2020). Notably, the MSCs present in different embryonic development stages, including postembryonic and postnatal tissues, constitute a population of sub-totipotent stem cells or progenitors, which were recently defined as MSC systems, have been confirmed to have molecular heterogeneity at single-cell transcriptomic level. MSCs maintain tissue homeostasis in three main ways. First, the MSCs residing in the major tissues, including adipose, bone, cartilage, muscles, divide and differentiate into targeted cell types to support the expansion, regeneration, and homeostasis of these tissues (Hilgendorf et al., 2019). Second, MSCs residing in tissue perivascular niches interact closely with their surroundings, which harbor varied cell types and soluble factors that further influence MSC behavior (Crisan et al., 2008; Sui et al., 2020). Third, MSCs themselves also secrete abundant types of biofactors and extracellular vesicles (EVs) to potentially affect their surroundings, including supporting hematopoiesis and modulating immune responses (Wang et al., 2014; Kfoury and Scadden, 2015; Sui et al., 2020). These functional capabilities contribute to MSC modulation in tissue homeostasis. However, the regulation of MSC function in these processes is immensely complex and tightly controlled and warrants extensive studies.

Long non-coding RNAs (lncRNAs) are transcripts with an average length of >200 nucleotides, lack protein-coding potential, and were previously considered transcriptional noise (Djebali et al., 2012). Most lncRNAs are evolutionarily conserved and species-specific, albeit less conserved across species, and they have diverse biological functions (Jin et al., 2011). According to genome-wide association studies (GWAS), non-coding intervals cover over one-third of the phenotype-associated locations. Nevertheless, lncRNAs largely remain to be identified, and their association and their functions require intensive studies (Jin et al., 2011). With the development of high-throughput sequencing, microarrays, and bioinformatics, an increasing number of lncRNAs has been identified, and increasing evidence has confirmed their roles as master regulators of various biological processes, including the maintenance of MSC homeostasis and multi-differentiation functions through diverse mechanisms at the epigenetic, transcriptional, and translational level.

In this review, we provide an overview of the MSC characteristics and their contributions to tissue homeostasis, and highlight the role of lncRNAs in modulating MSC homeostasis and differentiation. We also discuss the challenges and perspectives underlying lncRNA usage in preclinical research and clinical application. We aim to elucidate the underlying mechanisms involved in this process, which could help provide promising targets for MSC-based therapies.

## MSCs Contribute to Tissue Homeostasis

Mesenchymal stem cells were first identified from bone marrow by Friedenstein et al. (1976) in the 1950s; thereafter, scientists revealed that they are present in almost all connective tissues, and can also reside in fetal or adult somatic tissues, including the amniotic membrane (Parolini et al., 2008), umbilical cord (Romanov et al., 2003), adipose tissue (Zuk et al., 2002), skin (Orciani and Di Primio, 2013), peripheral blood (He et al., 2007), dental pulp (Huang et al., 2009), fetal liver (Zhang et al., 2005), and synovial membrane (De Bari et al., 2001). The source tissue from which MSCs are derived determines their differentiation potential (Xu et al., 2017). Bone marrow-derived MSCs (BMSCs) and adipose-derived MSCs (ADSCs) share similar morphological features and cell surface markers; however, many studies have indicated that significant biological differences exist, including differentiation potential. For example, BMSCs exhibit higher osteogenic but lower adipogenic differentiation capacity compared to ADSCs (Xu et al., 2017). ADSCs produce more neurosphere-derived neuron-like cells compared to BMSCs; therefore, ADSCs are a more suitable source for cell transplantation for treating spinal cord injury (Chung et al., 2013). Therefore, clarifying the intrinsic biological characteristic of MSCs derived from different sources and choosing the appropriate MSCs are important for their clinical application. To create a standard criterion for univocally defining the identity of MSCs used for scientific research and preclinical studies, the International Society for Cellular Therapy established the minimum criteria required for defining MSCs (Dominici et al., 2006; Wang et al., 2019): (1) MSCs must be fibroblast-like plastic-adherent cells when maintained in standard culture conditions; (2) ≥ 95% of the MSC population must express CD105, CD73, and CD90, and lack (≤2% positive) CD45, CD34, CD14 or CD11b, CD79a or CD19, and HLA-class II expression; (3) MSCs must have the capacity to differentiate into adipocytes, osteoblasts, and chondroblasts *in vitro*. Later studies have indicated that besides the capacity to differentiate into mesenchymal lineages, MSCs also have the potential to trans-differentiate into the unrelated germline ectodermal (neurocytes) and endodermal lineages (hepatocytes).

Recently, a new concept of MSC system was proposed by Wang et al. (2019), which was regarded as all MSCs derived from different stages of embryonic development, from postembryonic sub-totipotent stem cells to progenitors (Zhao, 2013). The MSC system well defined the important self-renewal and differentiation, immunomodulatory, and tissue homeostasis properties of MSCs, which provides a more comprehensive view of MSCs and better explains the heterogeneity of MSCs in differentiation potential and immunomodulatory functions. MSCs that reside in tissues such as bone marrow, adipose, cartilage, and muscle primarily form unique niches with a quiescent state. When exposed to stimulus such as injury, inflammation, and medicine, MSCs enter an active state to divide and differentiate into specialized cell types to support the expansion and homeostasis of these tissues (Méndez-Ferrer et al., 2010; Hilgendorf et al., 2019; Hu et al., 2020). Besides, MSCs interact closely with their surroundings by secreting variable biofactors and EVs to support hematopoiesis and modulate immune responses; the surrounding niches in which MSCs reside also influence their behavior (Crisan et al., 2008; Zhao et al., 2014). For example, during wound healing, skin residential ADSCs divide and migrate to injured sites and differentiate into skin cells such as dermal fibroblasts (DFs) to replace and regenerate damaged cells. On the other hand, ADSCs activate wound healing via the autocrine and paracrine pathways. Together with other skin cells such as DFs, ADSCs secrete factors to form the extracellular matrix and interact with each other to promote wound healing, maintain skin structure, and modulate skin homeostasis (Mazini et al., 2020). Another example is BMSCs, which express nestin, in the perivascular stroma, can self-renew and differentiate into osteochondral lineages that form a unique niche in the bone marrow to maintain hematopoietic stem cell (HSC) homeostasis, such as modulating HSC proliferation, differentiation, and recruitment (Méndez-Ferrer et al., 2010). In the endosteal niche, these BMSCs, together with osteoblasts, maintain HSCs in a quiescent state. When subjected to injury, MSCs expressing LepR and Gli1 divide and contribute to bone repair and regeneration (Zhou et al., 2014; Shi et al., 2017). In lethally irradiated mice, the injection of MSCs deficient in nestin expression notably reduced HSC homing to the bone marrow (Méndez-Ferrer et al., 2010). BMSC dysfunction, including aberrant proliferation and differentiation, is the crucial pathogenesis of bone degeneration and hematopoiesis suppression. Moreover, MSCs are indispensable in maintaining the homeostasis of other tissues, including intestinal (Stzepourginski et al., 2017) and skeletal muscle (Wosczyna et al., 2019).

**FIGURE 1:**
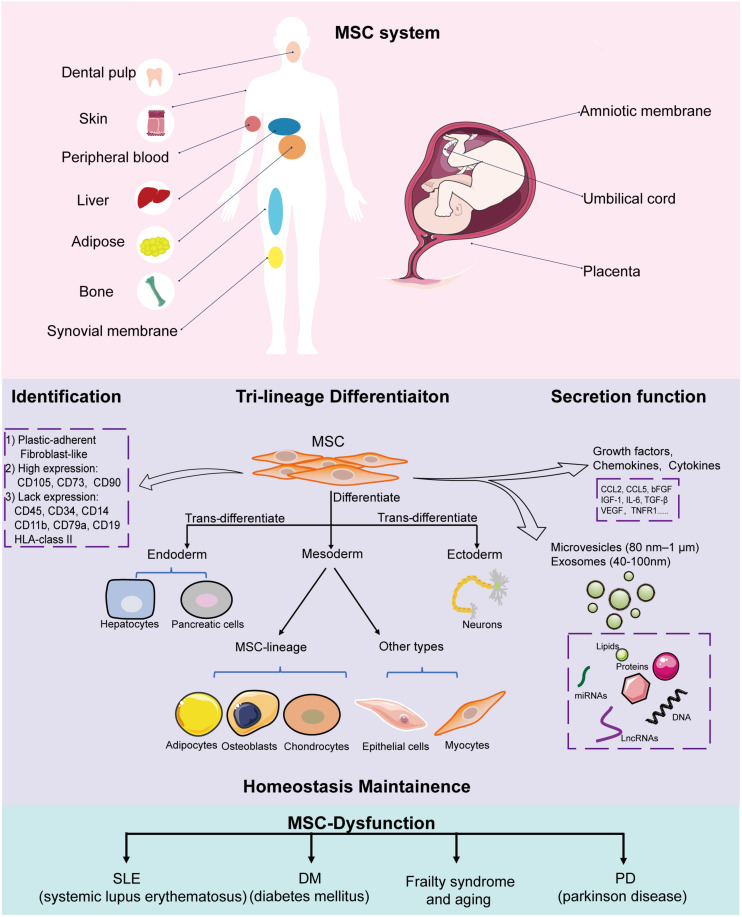
Implications of MSCs in homeostasis. MSCs can be isolated from a variety of tissues, including the amniotic membrane, umbilical cord, placenta, adipose tissue, skin, peripheral blood, dental pulp, and fetal liver. All MSCs derived from different stages of embryonic development, from postembryonic sub-totipotent stem cells to progenitors, are defined as MSC systems. During *in vitro* culture, MSCs must: (1) be fibroblast-like and plastic-adherent; (2) express CD105, CD73, and CD90, and lack CD45, CD34, CD14 or CD11b, CD79a or CD19, and HLA class II expression; (3) differentiate into adipocytes, osteoblasts, and chondroblasts. MSCs have trilineage differentiation potential, can secrete bioactive molecules and EVs (microvesicles and exosomes) to help tissue repair and maintain homeostasis. MSC dysfunction leads to disease-related MSC alterations that induce homeostasis disorder systemic disease.

So far, there have been breakthroughs in understanding the biological characteristics and potential therapeutic values of MSCs. In general, MSCs have multi-directional differentiation potential, can secrete bioactive molecules to migrate and home to injured or inflamed sites, and have powerful immunomodulating ability, thereby making them important contributors in tissue repair and homeostasis maintenance (Figure 1) (Vizoso et al., 2019; Wang et al., 2019; Bulati et al., 2020).

### MSCs and Their Multilineage Differentiation Potential in Tissue Repair and Homeostasis

Mesenchymal stem cells maintain tissue homeostasis based on their differentiation potential by serving as a source of renewable progenitor cells to repair injured tissues and replace cells in routine cellular turnover throughout adult life (Spees et al., 2016; Chen et al., 2017). MSCs are adult stem cells that present in many tissues and can differentiate into multiple mesenchymal lineage cell types such as adipocytes, osteoblasts, chondrocytes, and myoblasts under specific culture conditions (Boeuf and Richter, 2010; Scott et al., 2011; Westhrin et al., 2015; Chen et al., 2018). Besides, when exposed to certain extracellular cues, MSCs can also give rise to cross-lineage cell types like endodermal-hepatocyte and ectodermal-neurons, which is also known as trans-differentiate potential (Song and Tuan, 2004).

During bone tissue fracture, MSCs are recruited to the injury site and differentiate into osteoblasts to aid the repair and reconstitution of injured bone tissue (Freitas et al., 2019; Moura et al., 2020). MSCs can differentiate into cardiac cells under specific conditions *in vitro*; genetically manipulated MSCs with Akt1 and Wnt11 overexpression exhibit enhanced cardiac differentiation as verified by the elevated cardiac markers Nkx2.5, GATA4, α-MHC, and BNP, indicating that the transplantation of genetically engineered MSCs is a promising strategy for treating acute myocardial infarction (Chen et al., 2018). Moreover, MSCs also have the potential to trans-differentiate into endoderm and ectoderm cells to help repair specific tissues and organs. MSCs induced by chemically defined media containing specific cytokines and growth factors *in vitro* can trans-differentiate into hepatocyte-like cells with the functional properties of albumin synthesis and secretion, cytochrome P450 enzyme activity, glycogen storage, urea biosynthesis, and the expression of hepatocyte-specific genes (He et al., 2013; Fu et al., 2016; Maymó et al., 2018; Furuya et al., 2019), and can reconstitute liver function *in vivo* in experimental hepatic injury murine models (Xu et al., 2014; Fu et al., 2016). MSCs also have the capacity to produce pancreas-like cells under stepwise induction by cytokine cocktails (Yu et al., 2015; Mehrfarjam et al., 2016), via pancreatic extract or coculture with pancreatic adult stem cells (Lee et al., 2008; Hefei et al., 2015). MSC-derived insulin-producing cells express pancreatic β cell-related genes, respond to glucose challenge *in vitro*, and have the potential to improve glucose tolerance in diabetic 90% pancreatectomy rats *in vivo* (Yu et al., 2015). Further, MSCs can tans-differentiate into endothelial cells with the endothelial phenotype and express endothelial nitric oxide synthase, which contributed to improving endothelial function in a vascular injury rat model (Jiang et al., 2006; Yue et al., 2008).

Although the multi-differentiation capacity of MSCs ensures their tissue repair and regeneration function, the increasing application of MSCs clinically has reported that only a small amount of MSCs undergo subsequent differentiation into the targeted cell type after transplantation while still receiving functional improvement (Ferrand et al., 2011; Lai et al., 2015; Vizoso et al., 2019). Other mechanisms may confer MSCs efficacy in damaged tissues and the maintenance of tissue homeostasis.

### The MSC Secretome in Tissue Homeostasis

Increasing evidence supports the idea that intravenously injected MSCs can home specifically to sites of ischemia, damage, or inflammation, while not requiring induction into a specific functional cell type in advance (Price et al., 2006; Ye and Zhang, 2017; Ben Menachem-Zidon et al., 2019). Yet, other studies have shown poor survival and transient retainment of transplanted MSCs within the host tissue (Yeo et al., 2013; Miao et al., 2017), indicating that MSCs may not exert their therapeutic effects directly; rather, it occurs through the secretion of bioactive factors to provide a conducive microenvironment to facilitate the repair and regeneration of injured tissues.

Mesenchymal stem cells with the potential for synthesizing and secreting a variety of bioactive factors (e.g., cytokines and chemokines), and to affect nearby cells were first described by Haynesworth et al. (1996). In 2009, Bruno et al. (2009) reported that a new form of MSC secretion, termed microvesicles (80 nm to 1 μm), was protective against acute tubular injury. The next year, Lai et al. (2010) demonstrated a specific class of extracellular vesicles (EVs) with a diameter of 40–100 nm, defined as exosomes. The multiple bioactive factors, together with the EVs (e.g., exosomes and microvesicles), are generally referred to as MSC secretome. Subsequent studies reported that the MSC secretome has important effects in promoting angiogenesis, modulating immunity, and hematopoietic support (Lai et al., 2015; Konala et al., 2016). The composition of the soluble factors of MSCs derived from different tissues may vary, but they often secrete cytokines (e.g., CCL2, CCL5, bFGF, IL-6, TGF-β, and VEGF), contributing to tissue development, cell differentiation, and tumor growth and metastasis (Wang et al., 2019). Some factors (e.g., IL-6, IL-10, PGE2, HGF, nitric oxide, and human HLA-G) account for the immunomodulatory functions of MSCs (Wang et al., 2019). MSCs can also secrete neurotrophic factors, such as brain- and glial-derived neurotrophic factors (e.g., nerve growth factor), making them attractive cellular sources for brain disorders (Lopatina et al., 2019). Moreover, MSC-derived EVs also exhibit tissue repair and immunomodulation functions. Our group demonstrated that MSC-derived exosomes can promote the angiogenesis of human brain microvascular endothelial cells and contribute to alleviating Parkinson disease (PD) in a mouse model (Xue et al., 2021). Further, MSC exosomes inhibited inflammatory responses and reactive astrogliosis *in vitro* and *in vivo*, and repaired learning and memory impairments induced by status epilepticus in a mouse model (Xian et al., 2019). In an allogeneic hematopoietic stem cell transplantation animal model, EVs derived from human umbilical cord-derived MSCs prevented acute graft-versus-host disease (GVHD) (Wang et al., 2016).

## Overview of lncRNAs

Accumulating evidence supports the role of lncRNAs as master regulators of various biological processes, including the maintenance of MSC homeostasis and multi-differentiation functions through diverse mechanisms. The recent development of genome technology opened the door to understanding their functional importance. Conventionally, lncRNAs are transcribed by RNA polymerase II, containing multi-exons, processed by alternative splicing, 3′ polyadenylated and 5′ capped, and present transcriptional activation activity like that of mRNAs (Djebali et al., 2012; Ma et al., 2013; Lagarde et al., 2017). Although lncRNAs are distributed widely across species, they are poorly conserved and exhibit low expression levels, making them species-specific features and easily regarded as transcriptional noise (Ma et al., 2013). Moreover, lncRNAs exhibit a spatiotemporal and cell-, tissue-, and development-specific expression pattern (Shi et al., 2020), and their subcellular location in the nucleus or the cytoplasm determines their functions and working mechanisms (Chen, 2016). Nuclear lncRNAs are usually involved in transcriptional regulation, including interaction with chromatin regulation and RNA processing. Cytoplasmic lncRNAs tend to affect translation, such as modulating mRNA stability and cellular signaling cascades (Schmitt and Chang, 2016).

**FIGURE 2:**
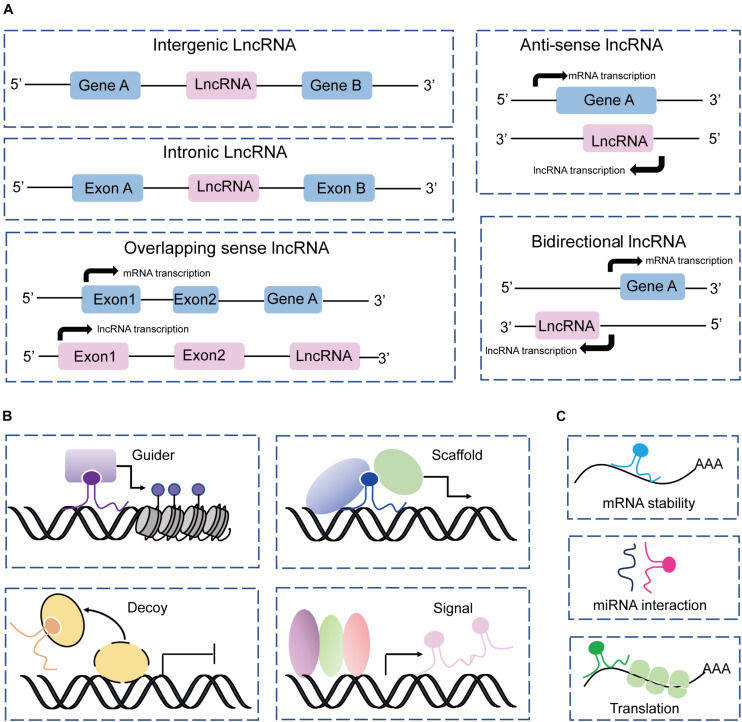
Classification and mechanism of lncRNAs. **(A)** Classification of lncRNAs according to the protein-coding genes as intergenic, intronic, sense, antisense, and bidirectional. **(B)** LncRNAs acting as guides, scaffolds, decoys, and signals to perform their functions with DNAs and proteins. **(C)** LncRNAs interact with RNAs to regulate RNA stability, and translation, and sponge miRNAs.

Based on the genome location of protein-coding genes, lncRNAs can be classified into five groups: intergenic, intronic, sense, antisense, and bidirectional (Ma et al., 2013; Jarroux et al., 2017; Fernandes et al., 2019), which are described in Figure 2A. This classification is widely used by the GENCODE/Ensemble database in the annotation of transcript biotypes, as well as newly assembled lncRNA transcripts identified by laboratories. Initially, lncRNA transcripts can be classified as either intergenic or intragenic; the intragenic lncRNAs overlap with coding genes and are further classified into antisense, bidirectional, intronic, and overlapping sense lncRNAs. Additionally, lncRNAs commonly perform their gene expression regulatory functions by acting as signals, decoys, guides, and scaffolds through main mechanisms by interacting with DNA, protein, and RNA (Wang and Chang, 2011; Schmitt and Chang, 2016), as illustrated in Figures 2B,C. However, the mechanisms underlying lncRNA regulation of gene expression and biological processes are complex and not simply confined to one archetype as we have summarized, and await more extensive discoveries.

Accumulating studies have implicated lncRNAs as vital regulators of variable bioprocesses, including genomic imprinting, chromosome modification, transcriptional interference, cell cycle, proliferation, immunobiology, and differentiation (Bartolomei et al., 1991; Quinn and Chang, 2016; Yang et al., 2018). In terms of the important biological functions of lncRNAs, dysregulation such as overexpression, deficiency, or mutation is suspected in the occurrence and progression of many diseases, including autoimmune disease, cardiovascular disease, and cancer (Batista and Chang, 2013; Beermann et al., 2016; Atianand et al., 2017). Moreover, emerging evidence has confirmed the contribution of lncRNAs in MSC differentiation, homeostasis, and related diseases (Tye et al., 2015); clarifying the roles and innate mechanisms of MSC-related lncRNAs in homeostasis will help provide promising targets for MSC-based therapies.

## MSC-Associated lncRNAs in Differentiation and Homeostasis

Mesenchymal stem cell differentiation is intricately regulated by multiple factors, including transcriptional factors (Runx2, PPARγ, MyoD, and GATA6), growth factors (VEGF, HGF, and EGF), and epigenetic factors such as DNA methylation, histone modification, RNA modification, and non-coding RNAs (miRNAs and lncRNAs) (Almalki and Agrawal, 2016; Sui et al., 2020). Recent studies have shown that lncRNAs are relatively new differentiation regulators that exert their functions through variable mechanisms, and await extensive studies. Herein, we mainly focus on the MSC-associated lncRNAs in differentiation and homeostasis.

### LncRNAs in MSC-Derived Multilineage Differentiation

**FIGURE 3:**
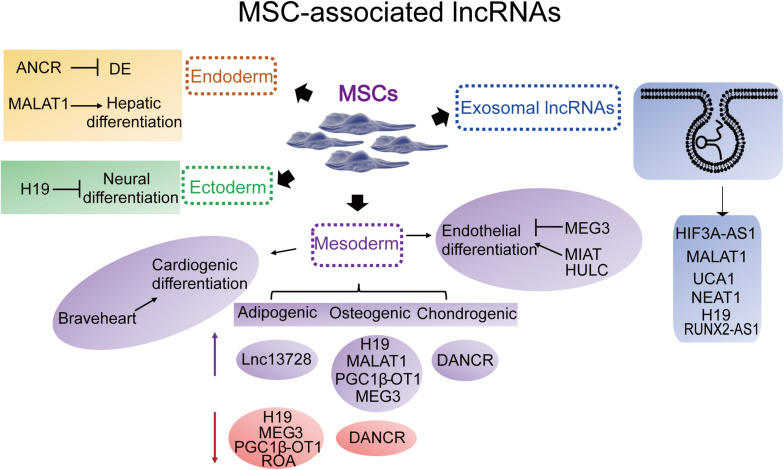
Representative MSC differentiation- and exosome-associated lncRNAs. Endoderm: ANCR (DANCR), (differentiation) antagonizing non-protein coding RNA; MALAT1, metastasis-associated lung adenocarcinoma transcript 1. Ectoderm: H19. Mesoderm-cardiac: Braveheart. Mesoderm-adipogenic: PGC1β-OT1, peroxisome proliferator-activated receptor γ coactivator-1β-OT1; lnc13728; H19; MEG3, maternally expressed 3. Mesoderm-osteogenic: H19; MEG3; MALAT1; DANCR; PGC1β-OT1. Mesoderm-chondrogenic: DANCR. Mesoderm-endothelial: MEG3; MIAT, myocardial infarction-associated transcript; HULC, highly upregulated in liver cancer. Exosomal lncRNAs: HIF3A-AS1, HIF3A antisense 1; MALAT1; UCA1, urothelial carcinoma-associated 1; NEAT1, nuclear paraspeckle assembly transcript 1; H19; KLF3-AS1, KLF3 antisense 1.

Long non-coding RNAs involved in MSC-derived lineage (adipocytes, osteoblasts and chondrocytes) differentiation have been extensively studied while remaining relatively less well studied in other directions such as endoderm and ectoderm lineage commitment and differentiation. Herein, we provide an overview of the essential lncRNAs involved in MSC lineage commitment (Figure 3 and Table 1) and elaborate on the representative lncRNAs below.

**TABLE 1:** MSCs-associated lncRNAs in multi-lineage differentiation.

**Name of lncRNA**	**Function and MSC sources**	**Working model**	**Mechanism-effector and target**	**References**

**lncRNAs modulating MSC mesodermal lineage differentiation**				
**Osteogenesis, adipogenesis and chondrogenesis**				
H19	Inhibit adipogenesis and BMSC	Epigenetic modulation	CTCF/H19/miR-675/HDAC	Huang et al., 2016
	Promote osteogenesis and BMSC	Sponging	miR-141, miR-22/Wnt/β-catenin pathway	Liang et al., 2016
			miR-138/FAK pathway	Wu et al., 2018
MEG3	Promote osteogenesis and BMSC (MM patients)	Decoy	SOX2/BMP4	Zhuang et al., 2015
	Inhibit osteogenesis and DFSCs	Unclassified	EZH2/Wnt genes	Deng et al., 2018
	Inhibit osteogenesis and BMSCs (PMOP patients)	Sponging	miR-133a-3p	Wang et al., 2017
	Inhibit adipogenesis, promote osteogenesis, and ADSC	Sponging	miR-140-5p.	Li et al., 2017
DANCR	Promote chondrogenesis and SMSC	Sponging	miR-1305/Smad4 axis	Zhang et al., 2017
	Inhibit osteogenesis and BMSC	Unclassified	p38 MAPK pathway	Zhang et al., 2018
MALAT1	Promote osteogenic and BMSC	Sponging	miR-34c/SATB2	Yang et al., 2019
			*miR-143*/OSX	Gao et al., 2018
PGC1β-OT1	Inhibit adipogenesis, promote osteogenesis, and BMSC	Sponging	miR-148a-3p/KDM6B	Yuan et al., 2019
ROA	Inhibit adipogenesis and BMSC	Decoy	hnRNP A1/PTX3/ERK	Pan et al., 2020
13728	Promote adipogenesis and ADSC	Unclassified	ZBED3/Wnt/β-catenin pathway	Xu et al., 2021
**Endothelial and cardiac differentiation**				
MEG3	Inhibit endothelial differentiation and BMSC	Post-transcriptional modulation	FOXM1/VEGF	Sun et al., 2018
MIAT	Promote endothelial differentiation and BMSC	Sponging	miR-200a/VEGF	Wang et al., 2018
HULC	Promote epithelial and smooth muscle-like cell differentiation and ADSC	Unclassified	BMP9/Wnt–β-catenin/Notch pathway	Li Y. et al., 2018
Braveheart	Promote cardiogenic differentiation	Unclassified	Mesp1	Hou et al., 2017
**lncRNAs modulating MSC trans-differentiation into endoderm lineage**				
ANCR	Inhibit DE commitment andADSC	Scaffold	ID2/PTK2B	Li et al., 2019
MALAT1	Promote trans-differentiate into hepatocyte and BMSC	Sponging	β-catenin/miR-217/ZEB1	Tan et al., 2019
**lncRNAs modulating MSC trans-differentiation into ectoderm lineage**				
H19	Inhibit trans-differentiate into neural-like cells and BMSC	Sponging	miR-675/IGFR	Farzi-Molan et al., 2018

#### LncRNAs in Mesodermal Lineage Differentiation

Mesenchymal stem cells tend to differentiate toward osteogenic, adipogenic, and chondrogenic lineages. Osteogenic and adipogenic MSC differentiation is a theoretically opposite process, during which the signaling pathways or transcription factors induced in adipogenesis occur at the cost of osteogenesis, and vice versa (Yuan et al., 2016). For example, peroxisome proliferator-activated receptor γ (PPARγ), a master regulator of MSC adipogenesis, and inhibits osteogenic differentiation. Bone morphogenetic protein (BMP) and Wnt, crucial inducers of MSC osteogenic differentiation, may hinder MSC adipogenic commitment by inactivating PPARγ. Many lncRNAs such as H19 and MEG3 act in the same manner. H19 is a paternally imprinted gene (Zhang and Tycko, 1992) that has been recently uncovered as an inhibitor during BMSC adipogenic differentiation through the epigenetic modulation of histone deacetylases (HDACs) (Huang et al., 2016). H19 also has the potential to promote MSC osteogenic differentiation by acting as a competing endogenous RNA (ceRNA) through sponging and inhibiting the expression of miR-22 and miR-141 (Liang et al., 2016). Similarly, H19 promotes tension-induced osteogenesis of BMSCs by sponging miR-138 and activates the downstream FAK pathway (Wu et al., 2018). Therefore, H19 is a key regulator in the multi-direction commitment of MSCs.

MEG3 is also an essential multi-functional regulator during MSC differentiation. During osteogenic differentiation, MSCs from patients with multiple myeloma (MM) had lower MEG3 expression compared to that from normal donors (Zhuang et al., 2015). MEG3 performs its function at multiple levels. At the transcriptional level, it may act as a decoy to dissociate SOX2 binding at the BMP4 promoter, repressing BMP4 expression, thereby transcriptionally activating BMP4 promotion of MSC osteogenic differentiation (Zhuang et al., 2015). MEG3 can also act as histone methylation mediators by binding to the enhancer of zeste homolog 2 (EZH2), which can inhibit the expression of Wnt pathway genes by inducing H3K27 trimethylation to inhibit the osteogenic differentiation of human dental follicle stem cells (hDFSCs) (Deng et al., 2018). At the post-transcriptional level, MEG3 may act as a ceRNA to regulate osteogenic gene expression, and its expression level is increased in postmenopausal osteoporosis (PMOP) patients as compared to that in healthy donors (Wang et al., 2017). During the osteogenic differentiation of BMSCs from PMOP, MEG3 may target miR-133a-3p to inhibit this process (Wang et al., 2017). In addition, MEG3 may control the balance between MSC adipogenic and osteogenic differentiation; its downregulation promotes adipogenic differentiation while inhibiting the osteogenic differentiation of human ADSCs via miR-140-5p (Li et al., 2017). Moreover, MEG3 is an inhibitor of the development of many bone disorders, such as bone tumors, osteoarthritis (OA), osteoporosis, RA, and ankylosing spondylitis (AS). These findings indicate that MEG3 may act as a novel target for diagnosing or treating such bone diseases (Sun et al., 2020).

DANCR was characterized as a differentiation-antagonizing lncRNA of progenitor cells (Kretz et al., 2012). It functions as a positive regulator of chondrogenesis of human synovium-derived MSCs (through the miR-1305–Smad4 axis) (Zhang et al., 2017) while acting as an inhibitor of periodontal ligament stem cell osteogenesis (Wang et al., 2020). Another study revealed that DANCR inhibited the osteogenic differentiation of human BMSCs through the p38–MAPK pathway (Zhang et al., 2018). The lncRNA MALAT1 is another well-known abundant and conserved imprinted gene that acts as a master regulator of osteogenic differentiation via the mechanism of sponging miRNAs such as miR-143 (Gao et al., 2018) and miR-34c (Yang et al., 2019). Another newly identified lncRNA, PGC1β-OT1, reciprocally modulates MSC adipogenic and osteogenic commitment by sponging miR-148a-3p and enhancing the effect of KDM6B (Yuan et al., 2019); the lncRNA ROA inhibits MSC adipogenic differentiation by destroying hnRNPA1 binding to the PTX3 promoter, thereby transcriptionally downregulating PTX3 and the ERK pathway (Pan et al., 2020). Moreover, our lab discovered that lncRNA13728 promoted ADSC adipogenic differentiation by upregulating ZBED3 and inhibiting the WNT–β-catenin pathway (Xu et al., 2021).

The disruption of the balance between MSC osteogenesis and adipogenesis leads to disorders such as osteoporosis (Hoshiba et al., 2012). Notably, lncRNAs such as MEG3 and PGC1β-OT1 reciprocally modulate MSC commitment to adipogenic and osteogenic cells; therefore, understanding the roles and underlying mechanisms of these lncRNAs may provide insights into improving the therapeutic method and effect of MSCs in diseases such as osteosarcoma, obesity, and OA.

In addition, MSCs can differentiate into mesoderm endotheliocytes and myocytes. The dysfunction of endothelial cell and myocyte generation leads to defects in angiogenesis and related cardiovascular disease. MEG3 inhibits BMSC endothelial differentiation by accelerating FOXM1 protein degradation via ubiquitination and decreasing VEGF expression (Sun et al., 2018). Moreover, the lncRNA MIAT, identified as a key contributor to development and disease, acts as a ceRNA of miR-200a and thereby targets VEGF to promote MSC endothelial differentiation (Wang et al., 2018). For MSC myogenesis, a recent study revealed that the lncRNA HULC promotes ADSC epithelial and smooth muscle-like cell differentiation by targeting BMP9, activating the Wnt–β-catenin pathway while inhibiting the Notch pathway (Li Y. et al., 2018). Another report showed the lncRNA Braveheart efficiently facilitates MSC cardiogenic differentiation by upregulating cardiac-specific transcription factors and epithelial-mesenchymal transition (EMT)-associated genes (Hou et al., 2017). Although MSCs have the potential to differentiate into all kinds of myocytes, functional lncRNAs in other types of myocyte commitment remain to be discovered.

#### LncRNAs in MSC Endodermal- and Ectodermal- Lineage Trans-Differentiation

Mesenchymal stem cells have tri-lineage differentiation potential; despite the mesodermal-lineage cells, MSCs can also trans-differentiate into ectodermal and endodermal lineages. Unlike the well-studied mesoderm lineage-associated lncRNAs described above, studies on the detailed functions of lncRNAs in MSC ectoderm and endoderm commitment are relatively rare (which are summarized in Table 1), and further exploration is warranted.

Generating definitive endoderm (DE) and its lineage hepatocytes is a prerequisite for cell replacement therapy for liver and pancreatic diseases as well as for drug testing and toxicology studies (Li et al., 2019). According to our findings, the lncRNA ANCR (DANCR) is an inhibitor during ADSC trans-differentiation toward DE, and the mechanism linked involves it acting as a scaffold to recruit PTBP1 to ID2 mRNA, enhancing the interaction between them and subsequently stabilizing the ID2 mRNA (Li et al., 2019). This finding reveals another function of ANCR in modulating MSC DE commitment besides regulating chondrogenesis and osteogenesis. MALAT1 also performs a function in MSC trans-differentiation into hepatocyte in addition to adipogenesis and osteogenesis. Tan et al. (2019) successfully induced BMSCs into hepatocytes using HGF *in vitro* and discovered that MALAT1 coordinated with β-catenin, sponging miR-217, and upregulating ZEB1 to enhance telomerase activity during MSC hepatic trans-differentiation.

Ectoderm lineage neural cells are the foundation of our nervous system; they are relatively difficult to generate *in vitro*. Generating abundant neural cells will help promote cell-based therapy for treating neurological disorders and nerve injuries. Many studies have demonstrated that MSCs have the potential to trans-differentiate into neural-like cells under specific stimulation, making them a novel therapy for treating nervous system diseases. A study that profiled lncRNAs during BMSC neural cell differentiation found that several lncRNAs were differentially expressed, suggesting their key roles in this process (Wu et al., 2015). A subsequent study confirmed that H19 has a negative effect on BMSC neural-like differentiation through the miR-675–IGFR axis (Farzi-Molan et al., 2018). In the future, the identification of new lncRNAs in MSC neurogenesis and studies of the extensive mechanisms involved, as well as *in vivo* experiments, are needed, which will contribute to improving MSC-based therapeutic effects in treating neurological disease.

These lncRNAs, i.e., DANCR, MALAT1 MEG3, and H19, represent a subset of lncRNAs that exert various functions through multiple mechanisms in specific cell types under specific stimulations, which subsequently attach MSC unique capabilities to meet the qualifications *in vivo* and for clinical usage *in vitro*.

### MSC Exosome-Derived LncRNAs and Their Implications in Clinical Usage

Increasing evidence suggests that the efficacy of MSC therapies is largely attributed to their paracrine secretion function, especially the exosomes (Dong et al., 2019). MSC-derived exosomes can shuttle a variety of bioactive molecules such as proteins, lipids, miRNA, lncRNAs, circular RNAs (circRNAs), and DNA to influence various bioprocesses, including development, immunity, and tissue homeostasis (Dong et al., 2019; Pegtel and Gould, 2019). Due to the advantages of low tumorigenic potential and low immunogenicity, exosomes are becoming novel, promising cell-free tools for tissue repair and diseases (Pegtel and Gould, 2019). Recently, functional lncRNAs derived from MSC exosomes have drawn increased attention, and some of these lncRNAs have been discovered. For example, the MSC exosomal lncRNA HIF3A-AS1 exhibits increased capacity in chondrocyte proliferation and cartilage repair in OA, which may be achieved through the miR-206–GIT1 axis (Liu et al., 2018a,b). Another study found that the exosomal lncRNA KLF3-AS1 alleviates cardiomyocyte pyroptosis and myocardial infarction through the miR-138-5p–Sirt1 axis (Mao et al., 2019). MALAT1 also resides in MSC exosomes; functional studies have shown that exosomal MALAT1 ameliorates osteoporotic by modulating the miR-34c–SATB2 axis (Yang et al., 2019) and can sponge miR-92a-3p and target ATG4a to fulfill its cardioprotective roles in doxorubicin-induced cardiac senescence and damage (Xia et al., 2020). Other exosomal lncRNAs such as UCA1 (Chen H. et al., 2020) and NEAT1 (Chen H. et al., 2020) also have a cardioprotective function by acting as ceRNAs.

The transfer of exosomes or microvesicles containing RNAs or other molecules between MSCs and the target cell type is one of the mechanisms by which MSCs perform their tissue repair functions (Spees et al., 2016). For example, H19 derived from MSC exosomes was transferred from MSCs to fibroblasts, thereby inhibiting fibroblast apoptosis and inflammation and activating the wound healing process in diabetic foot ulcers (Li et al., 2020). H19 could also be transferred to trophoblast cells via MSC-derived exosomes, enhancing trophoblast cell invasion and migration while inhibiting their apoptosis in preeclampsia (Chen Y. et al., 2020). Conversely, MSCs could also be the target cells during exosomal lncRNA transfer. MSCs derived from patients with MM had abundant exosomal lncRNA RUNX2-AS1; further studies revealed that it could be transferred from MM cells to MSCs and thereby prevent MSC osteogenesis by downregulating RUNX2 (Li B. et al., 2018), which provides a novel pathological mechanism of the bone lesion in patients with MM and could be a potential therapeutic target in the future.

These findings suggest that MSC-derived exosomes overexpressing lncRNAs such as H19 might be a novel direction for developing cell-free therapeutic strategies. Moreover, these exosomal lncRNAs are promising novel targets or biomarkers for treating and diagnosing diseases such as cardiomyopathy. In addition, understanding the tumor–stroma stem cell interactions, molecular transfer, and communication is also critical for developing novel and more effective strategies against cancer and other diseases.

## Concluding Remarks

Mesenchymal stem cells are key contributors in maintaining tissue homeostasis (Figure 1). The regulatory mechanisms underlying MSC functions are complicated, and are intricately regulated by multiple factors, i.e., transcriptional factors, growth factors, and epigenetic factors such as DNA methylation, histone modification, RNA modification, and non-coding RNAs (lncRNAs, miRNAs, and circRNAs). Recently, lncRNAs have emerged as prominent modulators of MSC fate commitment and functional homeostasis (Table 1) through variable mechanisms (Figure 2). Understanding the roles of lncRNAs in MSC functions in homeostasis will aid the development of promising targets for MSC-based therapies. However, issues and challenges remain to be investigated, including the conditions of MSCs used in basic research and clinical application, as well as the complex characteristics and mechanisms underlying lncRNA function.

## Challenges

As MSCs play an important role in tissue repair, regeneration, and homeostasis, their dysfunction may cause various systemic diseases. Clinical observation of allogenic MSC treatment of patients with autoimmune diseases, including systemic lupus erythematosus (SLE), diabetes mellitus (DM), rheumatoid arthritis (RA), and multiple sclerosis (MS) (Vizoso et al., 2019) indicates that the transplantation of external MSCs in good condition restores internal homeostasis. Further, MSC dysfunction indicates the onset of many diseases, including metabolic syndrome, DM, and RA, and aging syndromes such as Werner syndrome and Hutchinson–Gilford progeria syndrome (Liu et al., 2011; Zhang et al., 2015). Conversely, the continued inflammatory environment in these diseases may hinder MSC homing to the damage sites and probably result in MSC pool reduction and exhaustion (Shi et al., 2010), which contributes to the deterioration in MSC function and limits their use in autologous therapy.

To date, significant progress has been made in utilizing MSCs in basic preclinical research and clinical studies. However, some challenges should be overcome before the final clinical application (Wang et al., 2019). First, there is an urgent need for standard and consensus production (e.g., sources, medium, and culture conditions) to ensure the safety, reproducibility, and efficiency of MSCs administered to patients, which is also required in basic research. Second, MSCs derived from different tissues may have varying characteristics and functions; therefore, it is important to uncover the genetic background of different MSC sources and understand the specific innate characteristics of MSCs, which would aid the selection of the best seeds for fulfilling the specific clinical usage. Third, there is an urgent need to discover new genes or regulators such as the lncRNAs, as well as outstanding technologies to be developed to genetically modify MSCs and enhance their functions to boost their clinical application. Besides, the signals and mechanisms that modulate MSCs in tissue expansion, repair, and regeneration remain to be clarified, including the program that determines the balance between self-renewal and differentiation, the growth factors or signals that destroy the balance and trigger MSC expansion or differentiation, and how MSCs communicate with their surrounding niches to support a functional environment.

Mesenchymal stem cells maintain tissue homeostasis based on their differentiation potential to produce renewable progenitor cells to repair tissues and to replace cells in routine cellular turnover. MSCs tend to differentiate into mesenchymal lineage cells, while their trans-differentiation into endodermal and ectodermal lineage cells is limited. There are persistent challenges to fully understanding the underlying mechanisms in MSC differentiation, including identifying new signal and master transcription factors, and crosstalk between the signaling pathways involved in mediating and promoting MSC lineage differentiation and trans-differentiation rate. Manipulating MSCs with the overexpression of transcriptional factors increases their potential to differentiate into an intended cell type (Chen et al., 2018). However, a long journey remains before these genetically manipulated MSCs enter clinical application for treating diseases, unless safer methods are developed for manipulating MSCs with forced gene expression and to avoid activating the innate tendency of MSCs to differentiate into other unintended cell types.

Numerous lncRNAs participate in MSC lineage commitment, and lncRNAs derived from MSC exosomes exhibit enhanced tissue-protective and repair function. However, some challenges remain. On one hand, lncRNAs have multiple and varied functions and mechanisms of action, and lncRNAs largely remain unknown. e.g., H19 contributes to adipogenesis and osteogenesis, and resides in MSC exosomes to accelerate wound healing through different mechanisms. Moreover, lncRNAs may have an opposite effect on the same biological process, such as MEG3, which promotes and inhibits MSC osteogenic differentiation. First, the source of MSCs may confer the bidirectional effect on the lncRNA. lncRNAs usually display tissue- and spatiotemporal-specific expression patterns, and their aberrant expression is highly associated with disease and cancer occurrence. Therefore, lncRNAs may be differentially expressed at different stages of development, which confers their variable roles. Second, the MSC culture conditions *in vitro* may influence their stemness and functions, and the passage of MSCs used also matters. Therefore, as discussed above, there is an urgent need to establish a gold-standard approach for MSC basic research and clinical application. Taken together, extensive functional studies on one particular lncRNA can be performed in the future, and accompanying advanced molecular biotechnologies are being developed to better clarify and identify lncRNA targets and pathways and to screen for unknown lncRNA-interacting proteins. In addition, lncRNAs comprise a large proportion of the genome, and myriad functional lncRNAs remain to be discovered and studied. Moreover, most mechanisms of the existing studies on lncRNAs are focused on the downstream targets and pathways; the upstream stimulators and regulators that modulate lncRNA expression should be discovered. On the other hand, lncRNAs are poorly conserved among different species (Mirza et al., 2014), rendering it difficult or complicated to generate conditional knockout animal models to study the full function of lncRNAs, and complicating the development of lncRNAs as drug targets (Matsui and Corey, 2017). Despite these challenges, MSC-associated lncRNAs are promising targets and biomarkers for treating and diagnosing diseases. Nevertheless, opportunities coexist with challenges. There are emerging studies on lncRNA-based or -targeted drugs are emerging (Matsui and Corey, 2017), making them attractive therapeutic interventions in the future.

## Perspectives

Mesenchymal stem cell exosome-derived lncRNAs such as H19 shuttle between MSCs and fibroblasts to perform their function in facilitating wound healing in diabetic foot ulcers (Li et al., 2020), which indicates that MSC-derived exosomes with lncRNA overexpression might be a novel direction for developing cell-free therapeutic strategies and will improve MSC efficacy. With continued research in the future, genetically modified MSCs with improved tissue repair and regeneration functions will be achieved soon.

Over the last decade, non-coding RNAs (e.g., miRNAs and lncRNAs) have emerged as significant new therapeutic targets; many efforts have been dedicated to developing new oligonucleotide-based therapies aimed at promoting or antagonizing them. To date, over 100 antisense oligonucleotide (ASO)-based therapies have been developed and tested in clinical trials. The US Food and Drug Administration (FDA) has approved fomivirsen for treating cytomegalovirus retinitis and mipomersen for treating familial hypercholesterolemia (Adams et al., 2017). Unlike miRNAs, which are small and advantageous for delivering their mimics or inhibitors through synthetically modified oligoribonucleotides, lncRNAs are relatively large and usually are of a structured nature that makes it difficult to design effective mimics or inhibitors (Scacalossi et al., 2019). Although no clinical advances have been made with lncRNAs, they remain striking targets for clinical therapeutic intervention in the future. In addition, lncRNAs are relatively large and therefore more stable, rendering them suitable diagnostic and prognostic biomarkers for cancer. In recent years, it has been confirmed that circulating lncRNAs are valuable for detecting cancer types, as they are quite easily detected by common methods such as qRT-PCR, RNA sequencing (RNA-seq), and microarray in whole blood, plasma, serum, urine, saliva, and gastric juice samples; some circulating lncRNAs have been proven as sensitive biomarkers. More lncRNAs are being identified as diagnostic and prognostic biomarkers for varied diseases, especially for those caused by aberrant MSC dysfunction.

## Author Contributions

FZ and RZ conceived the project. YY, SL, CH, ZC, TL, LZ, and LW collected the data. YY wrote and revised the manuscript. FZ, HC, and RZ provided guidelines and edited the manuscript. All authors read and approved the final manuscript.

## Conflict of Interest

The authors declare that the research was conducted in the absence of any commercial or financial relationships that could be construed as a potential conflict of interest.
